# Pancreaticoduodenectomies with Concurrent Colectomies: Indications, Technical Issues, Complications, and Oncological Outcomes

**DOI:** 10.3390/jcm12247682

**Published:** 2023-12-14

**Authors:** Traian Dumitrascu

**Affiliations:** Division of Surgical Oncology, Department of General Surgery, Fundeni Clinical Institute, Carol Davila University of Medicine and Pharmacy, 050474 Bucharest, Romania; traian.dumitrascu@umfcd.ro; Tel./Fax: +40-213-180-417

**Keywords:** pancreaticoduodenectomy, colectomy, pancreatic cancer, colon cancer, morbidity, mortality, long-term outcomes

## Abstract

Multi-visceral resections for colon and pancreatic cancer (PDAC) are feasible, safe, and justified for early and late outcomes. However, the use of pancreaticoduodenectomy (PD) with concurrent colectomies is highly debatable in terms of morbidity and oncological benefits. Based on current literature data, this review assesses the early and long-term outcomes of PD with colectomies. The association represents a challenging but feasible option for a few patients with PDAC or locally advanced right colon cancer when negative resection margins are anticipated because long-term survival can be achieved. Concurrent colectomies during PD should be cautiously approached because they may significantly increase complication rates, including severe ones. Thus, patients should be fit enough to overcome potential severe complications. Patients with PD and colectomies can be classified as borderline resectable, considering the high risk of developing postoperative complications. Carefully selecting patients suitable for PD with concurrent colectomies is paramount to mitigate the potentially severe complications of the two surgical procedures and maximize the oncological benefits. These procedures should be performed at high-volume centers with extensive experience in pancreatectomies and colectomies, and each patient situation should be assessed using a multimodal approach, including high-quality imaging and neoadjuvant therapies, in a multidisciplinary team discussion.

## 1. Introduction

A pancreaticoduodenectomy (PD) is one of the most challenging abdominal surgical procedures and is associated with increased rates of postoperative complications, including severe ones, potentially leading to postoperative deaths. Even at very high-volume centers for pancreatic resections, the morbidity rates after PD are relatively high, while the perioperative mortality cannot be neglected. Specifically, in the experience in Verona of almost 3000 PDs over 20 years, the overall and severe morbidity rates were 59.9% and 20%, respectively, with postoperative pancreatic fistulas (POPFs) as a leading cause of morbidity (22.4%) and an in-hospital mortality rate of 2.3% [1]. Nationwide studies reporting outcomes of PD showed severe morbidity rates between 20.3% and 33% and in-hospital mortality rates between 1.3% and 9.8%. The centralization of pancreatic surgery has been associated with improvements in terms of failure to rescue, mortality, and re-admission rates [2,3,4,5,6]. Patient-level factors, such as advanced co-morbidities, male sex, and increased age, significantly contribute to increased mortality risks after PD [3].

A malignant periampullary pathology (with pancreatic ductal adenocarcinoma—PDAC as a leading indication), along with a benign pancreatic pathology, is most significant for patients with PD (87–96.4%). Other pathologies, including locally advanced colon cancer, represent an uncommon indication for PD [1,4,5,7].

Extended PD is required for specific pathologies (mainly borderline or locally advanced PDAC) to obtain negative resection margins. Considering that PD is a multi-visceral resection, in 2014, the International Study Group for Pancreatic Surgery defined standard and extended PDs to avoid confusion, particularly for multi-visceral resection and extended lymph node dissection [8,9]. Extended PD usually implies a venous resection (portal/superior mesenteric vein) [10]. In contrast, concurrent resections of the colon, small bowel, stomach, kidney, etc., are uncommon (less than 3% of PDs) [8,11,12,13,14]. Thus, a colon resection during PD is considered extended PD [8].

The en bloc resection of adjacent organs is sometimes required to resect patients with periampullary (particularly PDAC) or colon malignancies with curative intent. Based on the anatomical relationship, periampullary malignancies (with the PDAC as a leading cause) may extend to the mesocolon or colonic lumen. At the same time, a right or right-side transverse colon cancer may extend to the pancreatic head or duodenum. Multi-visceral (extended) resections for colon cancer and PDAC are feasible, safe, and justified for early and late outcomes [11,15,16,17,18,19,20]. However, the use of PD with concurrent colectomies is highly debatable because it may increase morbidity and mortality rates, while the oncological benefits are controversial.

PD and colectomies are surgical procedures with a high risk of morbidity and mortality. The two surgical procedures have specific postoperative complications, and it is widely considered that the severe morbidity and mortality rates for colectomies are far lower than those encountered in PDs. Interestingly, a recent study, based on analyses from the American College of Surgeons National Surgical Quality Improvement Program, showed increased morbidity rates for PDs compared with colectomies (38.5% vs. 26%) but similar 30-day mortality rates (2.7% vs. 2.8%) [21]. Although uncommonly performed, associations between two complex surgical procedures, such as PDs and colectomies, can potentially increase an operation’s complexity, morbidity, and mortality rates. Postoperative complications impact patients’ early outcomes and quality of life after PD/colectomies (slow postoperative recovery) and may harm oncological outcomes. Thus, severe postoperative complications correlate with delayed, incomplete, or even lower adjuvant chemotherapy rates in patients with PD for malignancies (mainly but not exclusively PDAC), which is a situation associated with increased recurrence rates and decreased survivals [22,23]. Furthermore, the development of significant morbidities after curative surgery for colon cancer is associated with increased recurrence rates and worse long-term survivals [24]. Thus, carefully selecting patients suitable for PD with concurrent colectomies is paramount to mitigate the potentially severe complications of the two combined surgical procedures and maximize the oncological benefits.

The present review aims to assess the indications, technical issues, and early and long-term outcomes of PD with concurrent colectomies based on data provided by the current literature.

## 2. Indications for PD with Concurrent Colectomies

As mentioned above, the association of PD with colectomies is an uncommon surgical procedure in clinical practice. Studies including 3275 to 24,421 PDs each showed that a concurrent colectomy was required in only 1.4–2% of the patients [12,25,26,27]. Other studies from single high-volume centers showed the association of colectomies with PD in 4.4 to 11.5% of patients [28,29,30]. A segmental or right colectomy is the most common type associated with PD [25,26,27].

The main indication in the most extensive series of patients with PD and concurrent colectomies was periampullary malignancies, with PDAC as a leading indication [12,14,26,27,28,29,30,31]. However, many single-center studies providing early and/or late outcomes after PD with concurrent colectomies for PDAC/other periampullary malignancies include a minimal number of patients [28,29,32]; only a few single-center studies include at least ten patient outcomes [14,30,33]. Few other studies review the outcomes from previously published series or multicentric national databases [12,26,27,31,34]. Nevertheless, about 181 PD patients with concurrent colectomies for PDAC were described in the literature till 2017 [34].

An exceptional clinical scenario is locally advanced right colon cancer involving the pancreas and/or duodenum, which is another potential indication for PD with concurrent right colectomy. A few studies showed that the association of PD with right colectomies was necessary for about 0.3–2.6% of patients operated on with curative intent for right colon cancer [35,36,37,38,39,40]. The first case of PD with associated right colectomy for locally advanced right colon cancer was reported in 1953 [41]. The most significant of published single-center series presenting the outcomes of PD with colectomies for locally advanced colon cancer includes a minimal number of patients [29,33,35,36,38,42,43,44,45,46,47,48,49,50,51]; only a few single-center studies include at least ten patient outcomes [37,39,40,52,53,54]. Few other studies review the outcomes of previously published cases or from multicentric national databases [26,31,53,55,56,57,58]. Nevertheless, about 81 patients with PD and concurrent colectomies for colon cancer were described in the literature till 2017 [53].

A malignant duodenum-colic fistula also represents a possible but rare indication of PD with concurrent colectomies. The main cause of malignant duodenum-colic fistula is colon cancer; duodenal cancer represent a rare cause of duodenum-colic fistula [49,54]. The first case of PD with associated right colectomy for a primary duodenal carcinoma with duodenum-colic fistula was reported in 1978 [59]. The benign etiology of the duodenum-colic fistula (with Crohn’s disease as a leading cause) appears slightly higher than the malignant one [49]. Proper differentiation of benign and malignant etiology for duodenum-colic fistulas is of utmost importance because the benign etiology requires less extensive surgical procedures than PD with concurrent colectomies [49].

Synchronous double cancers arising from the periampullary region and right/transverse colon represent another potential indication for PD with concurrent colectomies [31]. These patients are in exceptional situations where the surgical procedures (PD and colectomy) can be performed separately, not in an en bloc setting.

**Figure 1:**
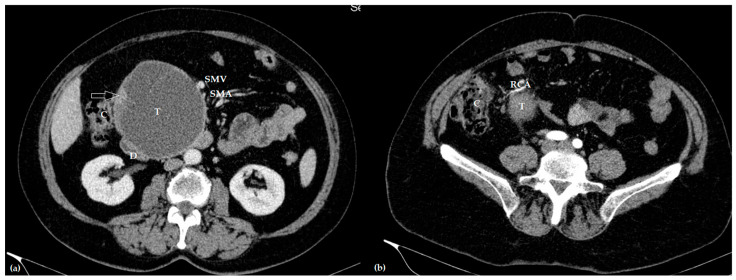
Contrast-enhanced axial computed tomography of the venous (**a**) and arterial (**b**) phase showing a large invasive intraductal papillary mucinous neoplasm (T), invading the duodenum (D) and right colic artery (RCA), and in close relationship with the superior mesenteric vein (SMV) and artery (SMA), and ascending colon (C) (the white arrow marks the malignant part of the tumor, without interface with the ascending colon).

**Figure 2:**
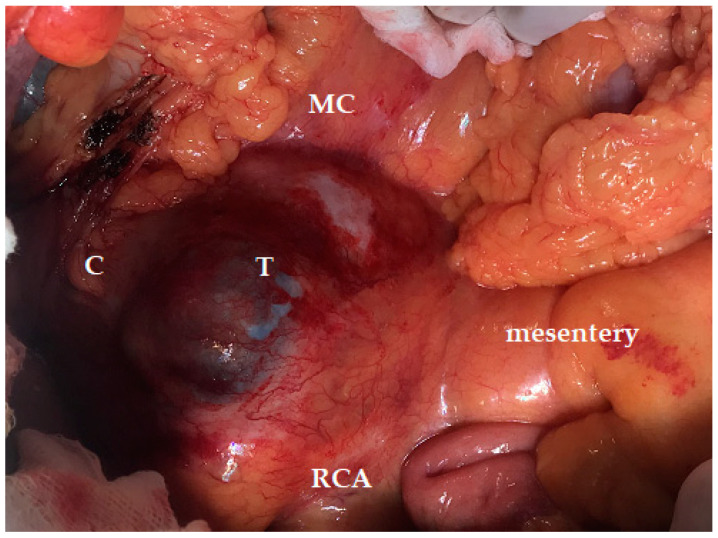
Intraoperative aspects of a large invasive intraductal papillary mucinous neoplasm (T), with infra-colic extension, invading the right and transverse mesocolon (MC) and the right colic artery (RCA) (C—the hepatic angle of the colon).

**Figure 3:**
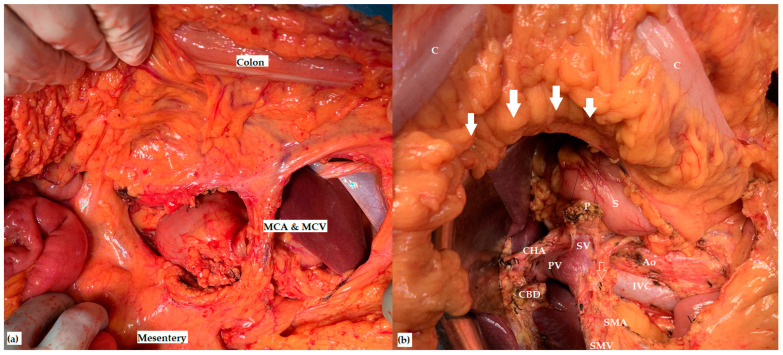
Intraoperative aspects after pancreaticoduodenectomy (**a**) with middle colic vessels preservation and (**b**) with middle colic vessels resection (MCA—middle colic artery; MCV—middle colic vein; CHA—common hepatic artery; CBD—common bile duct stump; PV—portal vein; SV—splenic vein; SMV—superior mesenteric vein; SMA—superior mesenteric artery; IVC—inferior vena cava; Ao—aorta; P—pancreatic remnant; S—the stomach; C—the transverse colon; the white unfilled arrow marks the MCA stump; the white filled arrows mark the preserved marginal colic arcade).

Nevertheless, associated colectomies are mandatory during PD whenever the ligation of the middle or marginal colic artery leads to colon ischemia [26,32] or for patients with an extensive invasion of the right or transverse mesocolon (Figure 1 and Figure 2). Unsurprisingly, a PD may imply transverse mesocolon resection with or without middle colic pedicle preservation. Usually, there is no impact on the colon blood supply if the paracolic marginal arcade is preserved (Figure 3). However, in the presence of rare anatomical variants (less than 5% of cases), the marginal artery of the colon can be discontinuous at the cecum and the ascending colon (absence of communications between the ileocolic and right colic arteries) or at the level of left colic angle (absence of communications between the middle colic and left colic arteries). In this late situation, the remaining transverse colon toward the left colic angle will suffer ischemia and necrosis after right colectomy without an accessory middle colic artery [60]. Unplanned colectomies during PD represent a significant proportion of patients in a few series [33].

PD with en bloc colectomies for malignancies should be performed only whenever negative resection margins are anticipated. Assessing the local tumor invasion and determining the likelihood of a margin-negative resection is based on contrast-enhanced multidetector-row computed tomography with a pancreas protocol [61]. It is worth mentioning, however, that the apparent invasion of either a pancreatic tumor into the colon or a colonic tumor into the pancreas is not always confirmed by the final pathological examination of the operative en bloc PD with the colectomy operative specimen because sometimes there are adhesions between the two organs due to a local inflammatory reaction. The differential diagnosis between an actual tumoral invasion and an inflammatory response is not always possible preoperatively at the imaging assessment or even intraoperatively at the surgical operation. In both scenarios, an en bloc resection is recommended because dissection along the tumor is oncologically unsafe, as it is associated with high rates of early local recurrence [62]. A few studies confirmed histological tumor infiltration into adjacent organs in only 53.4–63.6% of patients with multi-visceral resection for locally advanced colon cancer, while the remaining percentage of patients exhibit only adhesions due to local inflammations [15,18]. Thus, apparent local tumor invasions should not discourage surgeons from proceeding to an en bloc resection because, although challenging, this procedure might benefit a few patients when a negative resection margin can be achieved. It is also worth mentioning that a study showed 95% true tumoral invasion into the duodenum/pancreas in patients with PD and concurrent colectomies for colon cancer [53].

In several patients with locally advanced right colon cancer and limited duodenum invasion (apart from the ampulla of Vater proximity), a partial duodenectomy can be an alternative to PD to obtain negative resection margins. A systematic review published in 2014 comparing right colectomies with PD vs. partial duodenectomy for locally advanced colon cancer showed similar results in terms of postoperative complications for both groups of patients. However, the long-term prognosis appears to be less favorable for the group of patients with partial duodenectomy, albeit the statistical significance was not reached [56]. Similar long-term outcomes were recently confirmed by a review published in 2023 [58].

In a US national study, the most significant published to date, presenting the indication and outcomes of 430 patients with PD and concurrent colectomies, pancreatic, ampullary, and duodenal malignancies represented 70.8% of patients, while among patients with colon cancers, they were only 6.2%; other indications included neuroendocrine tumors (5.8%) and benign pathologies (16.8%) [27]. In a Dutch national study of 50 patients with PD and concurrent colectomies, the two main indications were PDAC (46%) and colon cancer (16%) [26].

## 3. Technical Issues of PD with Concurrent Colectomies

It is widely accepted that extended PD is associated with longer operative times [12,13,14] and more significant blood loss [13,14] compared with standard PD. A few studies showed statistically significantly higher operative times in patients with PD and associated colectomies compared with patients with standard PD. However, no differences were observed in the estimated blood loss [25,27,30]. Longer operative times may increase the risk of intraoperative bacterial contamination [63] and severe morbidity after PD [25].

An associated portal/superior mesenteric vein resection was reported in about 25–26% of patients with PD and concurrent colectomies [26,27]. Similar venous resection rates are reported during standard PD for PDAC in many western centers [14,64]. However, a few studies found statistically significant higher rates of venous resection in patients with PD and concurrent colectomies (particularly for PDAC) compared with standard PD: 32% vs. 13% (*p* = 0.007) [30]; 25% vs. 15%, (*p* < 0.001) [27]. A venous resection during PD may increase the perioperative complications rates and harm long-term survival [14,64]. However, a recent meta-analysis found similar overall morbidity, mortality, and survival rates in patients with pancreatectomies with and without venous resections [65].

**Figure 4:**
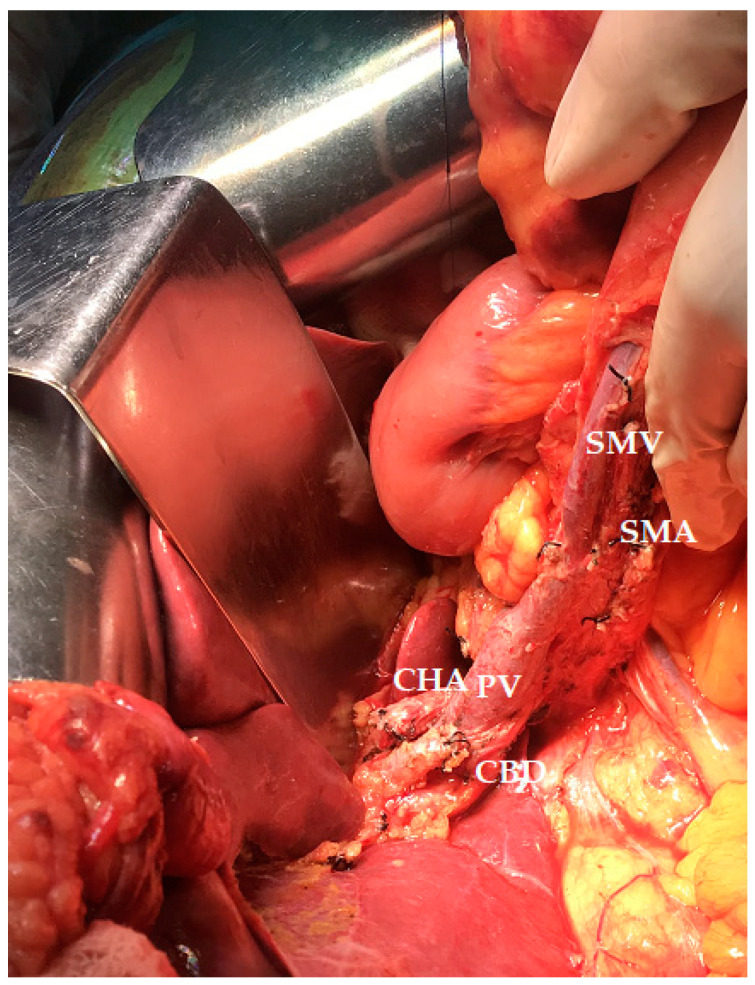
Intraoperative aspects after pancreaticoduodenectomy combining an artery-first approach with the Cattell-Braasch maneuver, showing excellent operative exposure (CHA—common hepatic artery; CBD—common bile duct stump; PV—portal vein; SMV—superior mesenteric vein; SMA—superior mesenteric artery).

For patients with large colonic tumors invading the pancreas or large pancreatic tumors invading the colon (Figure 1 and Figure 2), an infra-colic approach to PD appears to facilitate en bloc tumor resection, avoiding a non-curative resection [32,66,67]. This approach was first performed in 1981 by Nakao and coworkers [67]. An infra-colic approach may provide an excellent surgical field for tumors arising from the lower half of the pancreas or for large tumors with infra-colic development [68], and it may facilitate early diagnosis of superior mesenteric artery involvement [69] (Figure 2). Extensive mobilizing of the right colon and mesenteric root using the Cattell-Braasch maneuver is necessary to facilitate further en bloc resection [29,37]. Combining the Cattell-Braasch maneuver with the artery-first approach may facilitate en bloc venous resections during PD, providing good exposure to the operative field (Figure 4) and reducing the need for graft interposition [70]. It is worth mentioning that a few authors claim that artery-first approaches to PD, such as from the mesenteric and left posterior, may increase the risk of colonic ischemia and the need for unplanned concurrent colectomy during PD [31] because of excessive division of the middle colic artery [68].

Interestingly, a few authors have emphasized some potential pitfalls of en bloc PD with right colectomies. Thus, after transection of the proximal jejunum and distal ileum, it appears that there is a potential for improper identification of the two stumps. The authors propose completion of the colonic resection with ileo-colic anastomosis before transection of the jejunum to avoid any confusion [29].

A review published in 2018 showed that the most frequent type of colectomy associated with PD was right colectomy (84%), followed by segmental transverse colon resection (14%) [34]. In the most significant part of a previously published series of PD with associated colectomies, primary bowel reconstruction was the first option [25,26,27,28,29,30,34,35,36,39,40,50,54], while colostomy or ileostomy was uncommonly performed (up to 12% of the patients) [25,26,27,34].

Most patients with PD and concurrent colectomies reported in the literature had open surgeries. However, the minimally invasive laparoscopic approach for such complex procedures has recently been demonstrated as feasible and safe by highly experienced surgeons. However, experience with this approach is minimal [71,72,73,74].

## 4. Complications of PD with Concurrent Colectomies

POPF and anastomotic leaks are the primary clinically significant complications after PD and colectomies, respectively. The clinically relevant POPF rates after PD vary between 10.9% and 22.4% [1,4,5,7,75]; POPF represents the most common source of surgical mortality after PD. Nationwide population studies showed anastomotic leak rates after colectomies of 6.2% to 7% [76,77]. Recent multi-institutional studies demonstrated overall complications and anastomotic leakage rates after right colectomies of 15.9–38% and 5.6–7.4%, respectively, while the perioperative mortality is 2.1–6.1% [78,79]. Anastomotic leak rates of 1.9% after right colectomies in a study from Australia and New Zealand [80] are worth mentioning. Nevertheless, an anastomotic leak significantly increases the mortality risk after right colectomies [76,78,80].

A few systematic reviews showed an overall morbidity rate of 52.4–53.8% after colectomies with associated PD for locally advanced right colon cancer, which is the most frequent complication represented by POPF (23.8–27.5%) [53,57]. Another systematic review, including patients with PD and concurrent colectomies for PDAC, showed an overall morbidity rate of 25–91.3%, with perioperative mortality rates between 0% and 12% [34].

Several other studies reported overall morbidity and POPF rates of 12.5–100% [26,28,29,30,35,37,38,39,40,47,51,54,56] and between 7% and 100%, respectively [26,28,29,30,35,37,38,39,51,54], after PD with associated colectomies. Operative mortality rates of 2% to 17% were reported for patients with PD and associated colectomies [25,26,27,30,37,47,53,55]. The ileo-colic leak rate after PD with associated right colectomies is reported to be between 6% and 17% [26,30,34,47]. It is worth mentioning studies reporting nil ileo-colic leak and/or mortality rates after PD with associated right colectomies [28,29,36,37,38,39,40,44,50,51,54]. It appears that there are no differences in severe morbidity and mortality rates between patients with PD and concurrent colectomies for PDAC and locally advanced colon cancer, as a study showed [26].

Concurrent colectomies with PD in emergency settings are scarce but associated with exceptionally high mortality rates. A study from a very high-volume center and a recent systematic review showed that standard PD in emergency settings is associated with up to 40% mortality rates [81,82]. However, a few studies reported nil 90-day mortality rates even after colectomies with associated PD in emergency settings [40].

Extended PDs are widely considered to have increased severe morbidity and mortality rates, compared with standard PDs: 42.7–56.5% vs. 30.8–34.2% and 8.8–10.8% vs. 2.9–5.3%, respectively [12,14,83]. However, a few studies did not find any differences in the morbidity and mortality rates between standard PD and extended PD [13,84], albeit extended PD for PDAC was associated with worse survivals compared with standard PD [13,14]. Nevertheless, in a few studies, a colectomy was an independent predictor of mortality and/or severe morbidity in patients with PD [12,25,27,33,83]. Furthermore, a colectomy during PD was independently associated with an increased risk of overall morbidity and infectious complications [27].

Only a few studies assessed the outcomes of patients who underwent PD with or without associated colectomies. A study from Canada did not identify any statistically significant differences between the group of patients with PD and with or without associated colectomies for severe morbidity, POPF, delayed gastric emptying, or operative mortality rates (25% vs. 17%, 7% vs. 13%, 11% vs. 8%, and, respectively, 7% vs. 1%, *p* values ≥ 0.068) [30]. However, in the group of patients with PD and associated colectomies, the rate of severe hemorrhagic complications was statistically significantly higher than in the group of patients without associated colectomies (14% vs. 1%, *p* = 0.002) [30]. No differences in postoperative morbidity and mortality between the two groups of patients were observed in a Japanese comparative study, including mainly PDAC patients [28], and a study from Australia [32]. Another study from the American College of Surgeons National Surgical Improvement Program database comparing 159 patients with PD and associated colectomies with 10,965 patients with standard PD, including patients who underwent surgery between 2005 and 2012, found statistically significant differences between the two groups of patients for major morbidity (50.5% vs. 26.9%, *p* < 0.001), organ space infection (22.6% vs. 10.4%, *p* < 0.001), superficial surgical site infection (17.6% vs. 10.3%, *p* = 0.003), sepsis (22% vs. 10.2%, *p* < 0.001), septic shock (10.7% vs. 4.5%, *p* < 0.001), and 30-day mortality rates (8.8% vs. 2.8%, *p* < 0.001), and included a propensity-score matched analysis [25].

An updated analysis of the American College of Surgeons National Surgical Improvement Program database comparing 430 patients with PD and associated colectomies with 23,991 patients with standard PD, including patients who underwent surgery between 2014 and 2019, found statistically significant differences between the two groups of patients for overall morbidity (73% vs. 49%, *p* < 0.001), severe morbidity (68% vs. 42%, *p* < 0.001), clinically relevant POPF (22% vs. 16%, *p* = 0.004), any infectious complication (46% vs. 30%, *p* < 0.001), sepsis (21% vs. 12%, *p* < 0.001), septic shock (7% vs. 3%, *p* < 0.001), severe postoperative hemorrhage (44 vs. 18%, *p* < 0.001), unplanned reintubation and respirator dependence > 48 h (6% vs. 4%, *p* = 0.03, and 7% vs. 3%, *p* < 0.001, respectively), deep vein thrombosis (7% vs. 3%, *p* < 0.001), and re-laparotomy for complications rates (13% vs. 5%, *p* < 0.001). Interestingly, no statistically significant differences were observed between the two groups of patients in this late analysis for 30-day mortality rates (2% vs. 2.8%, *p* = 0.767) [27].

One explanation for the increased rate of infectious complications, sepsis, and septic shock in patients with PD and associated colectomies compared with standard PD might be related to the potential infectious complications of a colic/ileo-colic leak [25]. It is widely accepted that the colon has a higher bacterial load than the upper gastrointestinal tract [27]. Intraoperative bacterial contamination of the abdominal cavity during PD is associated with increased organ space, surgical site infection, and clinically relevant POPF rates [63]. The independent risk factors for abdominal contamination during PD are concurrent colectomies, internal biliary drainage, and longer operative time [63].

The improved mortality rates of both standard PD and PD with concurrent colectomies from the two extensive series analyses of the American College of Surgeons National Surgical Improvement Program database over time might be explained by the improvement of peri- and postoperative care of these patients, with increasing rescue-to-failure rates [27]. Thus, recent studies associated high-volume centers in pancreatectomies with significantly improved mortality, severe morbidity, and rescue-to-failure rates after PD [5,85].

Interestingly, the study by Harris and coworkers published in 2023 found statistically significantly increased rates of small Wirsung duct size and soft pancreatic texture in patients with PD and concurrent colectomies compared with the group of patients with standard PD [27]. This might explain, at least in part, the higher rates of clinically relevant POPF in the group of patients with PD and associated colectomies because small Wirsung duct size and soft pancreas are essential predictors of POPF formation after PD, widely used in recently proposed risk scores for POPF formation after PD [86,87]. Unfortunately, the study mentioned above [27] has no data about treating distal pancreatic stumps after PD. Recent studies have suggested the potential benefits of distal pancreatic stump anastomoses with the stomach over jejunum in patients with small Wirsung duct size and soft pancreatic texture [88,89].

A multicentric national database study compared the outcomes between patients with PD and concurrent colectomies and patients with only colectomies for colon cancer, showing statistically significantly higher rates of surgical site infections, wound dehiscence, and pneumonia in the associated PD group. Interestingly, no differences between the groups were observed for operative mortality (6.3% vs. 1.5%, *p* = 0.250) [55].

It is widely accepted that extended PD is associated with longer hospital stays than standard PD [12,14]. Statistically, significantly more extended hospital stays were reported for patients with PD and associated colectomies compared with standard PD in a few studies [25,27]. In contrast, no differences were reported in other studies [28,30].

## 5. Oncological Outcomes of PD with Concurrent Colectomies

A few studies showed that extended PD is associated with worse long-term survival than standard PD for PDAC [13,14]. Extracolonic malignancies appear to be associated with worse survival than locally advanced colon in cancer patients who underwent colectomies with associated PD [54].

A study from Canada, including mainly PD with associated colectomies for periampullary malignancies (with PDAC as a leading indication), showed negative resection margin rates of 93%, which did not have statistically significant differences from standard PD [30]. Although the median overall survival was 37 months for patients with standard PD and only 15 months for patients with PD and associated colectomies, the difference did not reach statistical significance [30]. Other studies reported 20–100% rates for negative-resection margins and median survivals of 14 months to 49 months after PD with concurrent colectomies for PDAC [29,32,34]. A Japanese study from 2004 showed similar negative resection margin rates (37.5% vs. 60.7%, *p* = 0.422) and median overall survivals (14 months vs. 12 months, *p* = 0.735) in patients with PD for PDAC with and without concurrent colectomies [28]. A Dutch study showed a median overall survival of 21 months in patients with PD and concurrent colectomies for PDAC and negative resection margins; when adjuvant therapy was added, the median overall survival reached 37 months [26]. The reported median survivals after PD with concurrent colectomies for PDAC appear comparable to those reported after PD with a venous resection [10,90] or even standard PD [23,91]. One might suggest that the colon or mesentery invasion in patients with PDAC does not reflect an aggressive tumor biology but the disease geography [29]. Thus, en bloc resection of these patients appears to be justified from the oncological point of view.

A few systematic reviews, including 81 to 105 patients with locally advanced right colon cancer who underwent colectomies with associated PD, showed high negative resection margins rates (95.5–97.5%) and median overall survivals between 70 and 168 months [53,57]. The only prognostic factor independently associated with improved survival was the absence of lymph node metastases. Recurrence rates after such complex procedures were 42.9%; most recurrence sites were distant metastases [57]. It is worth mentioning that although 62.1% of patients received adjuvant chemotherapy, none received neoadjuvant therapy. Interestingly, in this study, no differences in survivals were observed for patients with colectomies and associated PD for locally advanced right colon cancer with or without adjuvant chemotherapy or concerning chemotherapy regimens [57].

High rates of negative resection margins can be obtained with multi-visceral resections for locally advanced colon cancer (93.1–100%), which is associated with long-term survival [15,54]. A few studies reported a median overall survival of 21 months to 76 months after PD with associated colectomies for locally advanced right colon cancer [26,36,37,38,39,40,43,45,46,48,49,51,52,58]. Factors associated with improved overall survival after PD with associated colectomies for locally advanced right colon cancer were well- and moderately differentiated tumors [39,53], the absence of lymph node metastases [39,52,53], and adjuvant chemotherapy [39]. Histological proof of pancreatic invasion [52] and severe postoperative complications [92] harm long-term outcomes after PD with concurrent colectomies for colon cancer.

The oncological outcomes of the studies mentioned above should be regarded with caution because the use of adjuvant chemotherapy and chemotherapy regimens was largely variable between the studies, including both patients with colon cancer and periampullary malignancies treated with PD and concurrent colectomies [26,27,28,29,30,34,35,36,37,38,39,40,48,49,50,51,52,53,54,56,57].

The use of neoadjuvant therapy for both locally advanced colon cancer and periampullary malignancies may potentially be of benefit for patients because it provides better control of local disease, downstaging and downsizing, and selection of patients with aggressive biological behavior, aiming to increase negative resection margins, recurrence rates, and overall survival rates [93,94].

It was suggested that patients requiring PD with associated colectomies should be classified as borderline resectable and using neoadjuvant therapies is highly recommended [25]. Current international consensus criteria for borderline resectable PDAC include tumors at high risk for margin-positive resections with upfront surgery (the anatomic definition) and patients with high risk for morbidity or mortality after surgery, albeit negative resection margins can be achieved (the conditional definition) [61]. Considering that PD with concurrent colectomies is at high risk of causing postoperative complications, this group of patients can be considered borderline resectable, according to the international definition.

A recent meta-analysis of randomized studies showed statistically significantly improved negative resection margin rates and overall survival of patients with pancreatectomies for borderline resectable PDAC with neoadjuvant therapies compared with up-front surgery [94]. For resectable PDAC, another meta-analysis showed significantly higher rates of negative resection margins and negative lymph nodes after neoadjuvant therapies than upfront surgery [95]. As mentioned earlier, negative lymph nodes appear to be an important determinant of survival after PD with concurrent colectomies [39].

For patients at high risk of developing postoperative complications after PD, a situation that may impact access to adjuvant therapy in patients with malignancies, a neoadjuvant treatment should be taken into consideration to improve oncological long-term outcomes [22,23]. Nevertheless, a study published in 2023 showed statistically significantly higher rates of malignancies with the use of neoadjuvant therapies in patients with PD and concurrent colectomies compared with the standard PD group of patients [27]. A neoadjuvant therapy should be strongly considered whenever the risk of positive resection margins is high with upfront surgery. It is worth mentioning studies showing the benefit of adjuvant therapy in improving overall survival even in delayed settings for patients developing postoperative complications after PD for PDAC (up to 24 weeks after PD) [96].

## 6. Conclusions

PD with associated colectomies represents an exceptional but challenging procedure that increases the complexity of this surgery. To date, there are no widely accepted indications for such procedures. However, colectomies with associated PD represent a feasible option for a few patients with PDAC and other periampullary malignancies or locally advanced right colon cancer when negative resection margins are anticipated because long-term survival can be achieved, particularly for locally advanced right colon cancer without loco-regional lymph node metastases (Table 1). Furthermore, in a relatively large number of patients, the apparent invasion is related to local inflammation, and it is not confirmed by the final pathology examination, which is associated with a favorable long-term prognosis with en bloc resection.

Concurrent resection of the colon during PD should be cautiously approached because the association of colectomies with PD may significantly increase complication rates, including severe ones. Thus, patients proposed for PD with concurrent colectomies should be fit enough to overcome potential severe postoperative complications. Patients with indications for PD and concurrent colectomies can be classified as borderline resectable, considering the high risk of developing postoperative complications. Carefully selecting patients suitable for PD with en bloc colectomies is paramount to mitigate the potentially severe complications of the two combined surgical procedures and maximize the oncological benefits. Such complex surgical procedures should be performed at high-volume centers with extensive experience in pancreatectomies and colectomies, and each patient situation should be assessed using a multimodal approach, including high-quality imaging technologies and neoadjuvant therapies, in a multidisciplinary team discussion.

There is an emerging role for neoadjuvant therapies for locally advanced colon and periampullary malignancies to select better patients for surgery to improve local disease control and overall survival, and further evaluation of PD with concurrent colectomies in this setting is warranted.

Nevertheless, it is worth mentioning that the most significant of the evaluated studies published in the literature addressing the topic of colectomies with associated PD included a limited number of patients with a selective and heterogeneous population and reflected either non-high volume or high-volume center experiences. Furthermore, the use of adjuvant and neoadjuvant therapies was largely variable. All these represent a limitation of the present review, and the conclusions should be considered cautiously for both early and late outcomes for clinical decision-making.

## Figures and Tables

**Table 1:** Studies published in the literature in the last 10 years (2014–2023), including at least 10 patients, providing early and late outcomes of pancreaticoduodenectomies with concurrent colectomies.

Author, Year, Country	N^o^ of Patients (Period)	Pathology	Overall Morbidity	Severe Morbidity (Grade 3-4 Dindo)	POPF	DGE	Colonic Anastomotic Leak	Mortality	Neoadjuvant Treatment	Adjuvant Treatment	Negative Margins	Median overall Survival	5-Years Survival Rate
**Original single-center studies**													
Temple, 2014 [30] Canada	28 patients (2000–2010)	Malignancies and benign diseases (PDAC–32%, colon cancers–7%)	NR	25%	7%	11%	7%	7.1% ^a^	22%	NR	93%	15 months	35%
Schwartz, 2017 [33] USA	26 patients (2006–2015)	Malignancies and benign diseases	65%	NR	NR	NR	NR	19% ^a^	NR	NR	NR	NR	NR
Yan, 2021 [39] China	19 patients (2010–2019)	Colon cancers	NR	42%	NR	0%	0%	0% ^b^	21.1%	79.1%	100%	76 months	66%
Chen, 2021 [40] Taiwan	30 patients (1994–2018)	Colon cancers	50%	16.7%	13.3%	20%	0%	0% ^a^	NR	NR	90%	NR	51%
Das, 2023 [54] UK	10 patients (2013–2020)	Colon and duodenum cancers	70%	10%	30%	0%	0%	0% ^a^	40%	40%	100%	37 months	83%
**Original multicentric studies**													
Harris, 2015 [25] USA	159 patients (2005–2012)	Malignancies and benign diseases	NR	50.3%	NR	NR	NR	8.8% ^a^	5.3%	NR	NR	NR	NR
Marsman, 2016 [26] The Netherlands	50 patients (2004–2014)	Malignancies and benign diseases (PDAC 46%, colon cancers 16%)	86%	54%	2%	27.5%	6%	8% ^a^	10%	40%	66% (PDAC 48% Colon cancers 100%)	PDAC—13 months (37 months with adjuvant therapy) Colon cancers—24 months	PDAC—14% Colon cancers—100%
Harris, 2023 [27] USA	430 patients (2014–2019)	Malignancies and benign diseases	73%	65%	22%	NR	NR	2% ^b^	30%	NR	NR	NR	NR
**Systematic reviews**													
Cirocchi, 2014 [56]	39 patients (1980–2012)	Colon cancers	NR	12.8%	7.7%	NR	NR	0% ^b^	NR	23.1%	NR	NR	52%
Solaini, 2018 [34]	181 patients (1994–2017)	PDAC	73%	NR	NR	NR	5.5%	10% ^b^	NR	NR	50.7%	18 months	19%
Li, 2018 [53]	81 patients (1990–2017)	Colon cancers	53.8%	NR	8.6%	17.4%	NR	3.7% ^b^	NR	42.5%	97.5%	70.4 months	55.2%
Khalili, 2019 [57]	106 patients (1980–2017)	Colon cancers	52.4%	NR	23.8%	17.5%	NR	NR	0%	62.1%	95.5%	168 months	66.3%

^a^ 90-day mortality; ^b^ 30-day mortality; POPF—clinically-relevant postoperative pancreatic fistula; DGE—clinically-relevant delayed gastric emptying; PDAC—pancreatic ductal adenocarcinoma; NR—not reported.

## Data Availability

Not applicable.
